# Proteomic Analysis of the Peri-Infarct Area after Human Umbilical Cord Mesenchymal Stem Cell Transplantation in Experimental Stroke

**DOI:** 10.14336/AD.2016.0121

**Published:** 2016-10-01

**Authors:** Dongsheng He, Zhuo Zhang, Jiamin Lao, Hailan Meng, Lijuan Han, Fan Chen, Dan Ye, He Zhang, Yun Xu

**Affiliations:** ^1^Department of Neurology, Affiliated Drum Tower Hospital, and; ^2^Jiangsu Key Laboratory for Molecular Medicine, Nanjing University Medical School, Nanjing 210008, China.; ^3^The State Key Laboratory of Pharmaceutical Biotechnology, Nanjing University, Nanjing 210008, China; ^4^Department of Gastroenterology, Children’s Hospital of Nanjing, Nanjing Medical University, Nanjing 210008, China; ^5^Jiangsu Province Stroke Center for Diagnosis and Therapy, Nanjing 210008, China; ^6^Nanjing Neuropsychiatry Clinic Medical Center, Nanjing 210008, China

**Keywords:** stroke, hUC-MSCs, proteomics, iTRAQ, transplantation, human

## Abstract

Among various therapeutic approaches for stroke, treatment with human umbilical cord mesenchymal stem cells (hUC-MSCs) has acquired some promising results. However, the underlying mechanisms remain unclear. We analyzed the protein expression spectrum of the cortical peri-infarction region after ischemic stroke followed by treatment with hUC-MSCs, and found 16 proteins expressed differentially between groups treated with or without hUC-MSCs. These proteins were further determined by Gene Ontology term analysis and network with CD200-CD200R1, CCL21-CXCR3 and transcription factors. Three of them: Abca13, Grb2 and Ptgds were verified by qPCR and ELISA. We found the protein level of Abca13 and the mRNA level of Grb2 consistent with results from the proteomic analysis. Finally, the function of these proteins was described and the potential proteins that deserve to be further studied was also highlighted. Our data may provide possible underlying mechanisms for the treatment of stroke using hUC-MSCs.

Stroke is a leading cause of disability in China as well as in Western countries. Unfortunately, although much attention has been given to it, there is still a lack of effective treatments. Cell-based therapy for stroke, frequently studied in recent years, has shown promising results. For example, ischemic stroke treated with mesenchymal stem cells (MSCs) could improve functional outcome in animal studies and clinical settings [1-7]. In animal studies, the neurological deficit induced by ischemic stroke is significantly improved while infarct volume is significantly attenuated after transplantation of MSCs. Similarly, a clinical trial conducted by Lee and colleagues showed that neurological deficits of stroke patients transplanted with autologous MSCs were improved without cell-related adverse events during the short-term and long-term follow up periods. Although the findings are encouraged, the molecular mechanisms remain obscure. Recent studies have documented that MSCs have the capacity to pass through the blood-brain barrier and migrate throughout the forebrain and cerebellum, wherein the MSCs may differentiate into neuronal-like cells. The functional property of MSCs-derived newborn neurons and the network between them remain to be unverified. Therefore, a hypothesis that MSCs may exert their function by producing trophic factors such as brain-derived neurotrophic factor (BDNF), vascular endothelial growth factor (VEGF), hepatocyte growth factor (HGF), nerve growth factor (NGF), basic fibroblast growth factor (bFGF, FGF-2), insulin growth factor-1 (IGF-1), and so on has been generated [8-10].

Human umbilical cord mesenchymal stem cells (hUC-MSCs) are MSCs derived from the human umbilical cord, which can be used without raising any ethnical concerns because this extra-embryonic tissue is often discarded. hUC-MSCs express specific surface antigens; positive for CD105, CD73 and CD90, and negative for CD45, CD34, CD14 or CD11b, CD79a or CD19, and HLA-DR surface molecules. Besides having multipotent differentiation potential, hUC-MSCs share a close ontogenetic relationship to embryonic stem cells (ESCs) and proliferate faster than adult MSC [11]. Our previous study [12] showed that hUC-MSCs intravenously injected into ischemic animals could significantly improve neurological function and reduce infarct volume within 30 min after middle cerebral artery occlusions (MCAO), along with reduced expression of pro-inflammatory cytokines, including IL-1, TNF-α, IL-23 and IL-17, and increased expression of the anti-inflammatory cytokine such as IL-10. Interestingly, these effects could be partially inhibited by the TGF-β neutralizing antibody.

In the present study, iTRAQ (isobaric tags for relative and absolute quantification) was used to analyze the difference of protein expression between the stroke and sham groups, with or without intravenous injection of hUC-MSCs.

## MATERIAL AND METHODS

### Animals and MCAO model

Six to seven week old male C57BL/6 mice (weighing between 25 and 30g) were provided by the Drum Tower Hospital Animal Center and used according to protocols approved by the Committee of Experimental Animal Administration of Nanjing University, China.

Mice were randomly allocated to groups as described below. The randomization procedure was performed by the RAND function in Office Excel 2010. We first assigned a random number (from 1-102) to each mouse. Next, the mice were sorted by the random numbers (in ascending order). They were then allocated to the 34 groups according to their locations in the ranked random numbers series. The number of mice in each experimental group is listed in Table 1.

**Table1:** The number of mice in each experiment group.

Experiment	Group	No. of mice
Proteomics	sham	1-3
	MCAO 24h	4-6
	MCAO 48h	7-9
	MCAO 48h + hUC-MSCs	10-12
QPCR	Sham 6h	13-17
	MCAO 6h	18-22
	MCAO + hUC-MSCs 6h	23-27
	Sham 12h	28-32
	MCAO 12h	33-37
	MCAO + hUC-MSCs 12h	38-42
	Sham 24h	43-47
	MCAO 24h	48-52
	MCAO + hUC-MSCs 24h	53-57
	Sham 48h	58-62
	MCAO 48h	63-67
	MCAO + hUC-MSCs 48h	68-72
	Sham 72h	73-77
	MCAO 72h	78-82
	MCAO + hUC-MSCs 72h	83-87
	Sham 1W	88-92
	MCAO 1W	93-97
	MCAO + hUC-MSCs 1W	98-102
ELISA	Sham 24h	43-47
	MCAO 24h	48-52
	MCAO + hUC-MSCs 24h	53-57
	Sham 48h	58-62
	MCAO 48h	63-67
	MCAO + hUC-MSCs 48h	68-72
	Sham 72h	73-77
	MCAO 72h	78-82
	MCAO + hUC-MSCs 72h	83-87
	Sham 1W	88-92
	MCAO 1W	93-97
	MCAO + hUC-MSCs 1W	98-102

Focal cerebral ischemia was induced by MCAO as previously described [13]. Briefly, after mice were anaesthetized by intraperitoneal injection of sodium pentobarbital (1%) at a dose of 45 mg/kg, a midline cervical incision was made under a dissecting microscope and then the right common carotid artery and external carotid artery were isolated. Next, a poly-L-lysine coated nylon monofilament thread was inserted through the external carotid artery and advanced into the internal carotid artery to occlude the origin of the middle cerebral artery (approximately 12 mm). During the procedure, a homeothermic blanket and water pads were used to maintain body and head temperatures at 37 ± 0.5°C. After 90 min of occlusion, the filament was withdrawn for reperfusion. Sham-operated mice were subjected to the same procedure without MCAO.

### Preparation and injection of hUC-MSCs

hUC-MSCs from passages 2 to 3 (Shenzhen Beike Stem Cell Engineering Institute) were used for this study [14]. 4 X 10^6^ hUC-MSCs was injected via the tail vein within 30min after ischemia/reperfusion (I/R). While the same volume of NS was used as control.

### Sample preparation

Mice were sacrificed at the indicated time points under deep anesthesia using halothane (0.3-0.5 ml per mouse). The brains were removed quickly and the cortical peri-infarct areas were harvested and stored at -80°C for further analysis. At indicated time points, the acquired cortex was divided into 2 parts for quantitative real-time PCR (qPCR) and ELISA respectively as shown in Table 1.

### iTRAQ

A detailed protocol for proteomics analysis was followed as previously described [15]. Briefly, iTRAQ labeling (Applied Biosystems) coupled with online two-dimensional Nano LC/MS/MS system (2D-nanoLC-MS/MS) (Agilent, Waldbronn, Germany) was used; protein (100 μg) from each sample was denatured, alkylated, and digested before being labeled with iTRAQ reagents and mixed. After being cleaned, desalted, and vacuum-dried, the mixed sample was analyzed using the On-line two-dimensional Nano LC/MS/MS on a Nano-HPLC system coupled to a hybrid Q-TOF mass spectrometer (QSTAR XL, Applied Biosystems) equipped with a Nano-ESI source (Applied Biosystems) and a Nano-ESI needle (Picotip, FS360-50- 20; New Objective Inc., Woburn, MA). The ProteinPilot™ Software 3.0 (revision 114732) was used to analyze the differential expressed protein. A ratio more than 1.5-fold or less than 0.66-fold was deemed significant. Using the Software DAVID (http://david.abcc.ncifcrf.gov/) coupled with wego (http://wego.genomics.org.cn/cgi-bin/wego/index.pl), the GO term analysis was completed and the differential expressed protein of each group was determined. STRING (V9.1) software was then used to evaluate the network with crosstalk key molecules and with transcription factors.

### qPCR

Real-time PCR was performed as described previously [14]. Trizol reagent (Takara, life technologies, USA) was used to extract total RNA that were then reverse-transcribed into cDNA using a Prime Script RT reagent kit (Takara, Clontech,USA) for quantitative PCR (Takara, Clontech, USA) in the presence of a fluorescent dye (SYBR GreenI; Takara, Clontech, USA; n=5 per group). The relative level of mRNA was calculated and presented after normalization to glyceraldehyde-3-phosphate dehydrogenase ribosomal RNA.

The primer sequences used (Invitrogen, USA) are as follows:

Abca13 primers:
  Forward: 5-‘CCTGCCCTATGTGGTCCTGT-3’
  Reverse: 5’-TTCCCTTCCTCCTGTCCTTCC-3’
Grb2 primers:
  Forward: 5’-AGAATGGAAGCCATCGCCAA3’
  Reverse: 5’-CTGCACATCATTTCCAAACGGA3’
Ptgds primers:
  Forward: 5’-CAGTGGTGGAGGCCAACTAT-3’
  Reverse: 5’-CCAGCCCTCTGACTGACTTC-3’

### ELISA

The protein level of Abca13, Grb2 and Ptgds in all groups (n=5 per group) were detected using mouse cytokine ELISA kits (R&D Systems, Minneapolis, MN, USA) as described by the manufacture [14]. A microplate reader (Bio-Rad Laboratories, Hercules, CA) was used to read the optical density at 450nm.

### Statistics

Data were expressed as mean?±?SD. The comparisons among groups were determined by one-way analysis of variance (ANOVA) followed by Bonferroni’s *post hoc* test. All data analyses were conducted with the SPSS 17.0 software package (SPSS, Inc., Chicago, IL, USA). A value of *p*<0.05 was considered to be statistically significant.

## RESULTS

### Alterations in protein expression

The cortical peri-infarct areas were harvested at 24 h and 48 h after ischemic stroke with or without treatment of hUC-MSCs. Changes in protein expression were compared between groups.

**Table 2:** Differentially expressed proteins in each group

	mcao-24h/sham	mcao-48h/sham	mcao-48h/mcao-24h	mcao-48h + hUC-MSCs/mcao-48h
Up-regulated protein	Abca13,Ptgds, Hbb-b1	Abca13	Acot11,Acat2,Grb2,Scp2, Ppp2r5d,Rps12-ps11	Rrbp1, Anxa6, Slk
Down-regulated protein	Acot11,Acat2, Grb2,Scp2, Anp32b, Ppp2r5d	Acot11,Vps13d, Anp32b	Vps13d,Ptgds, Hbb-b1	Nup205, Psmd6

Compared to sham group, there were 3 up-regulated and 6 down-regulated proteins at 24 h after cerebral ischemia; there were 1 up-regulated and 3 down-regulated proteins at 48 h after cerebral ischemia. Further analysis showed that there were 6 up-regulated and 3 down-regulated proteins at 48 h after cerebral ischemia compared with the 24 h after cerebral ischemia group. There were also 3 up-regulated and 2 down-regulated proteins in the groups treated with hUC-MSCs compared with the 48 h after cerebral ischemia group.

**Figure 1.**
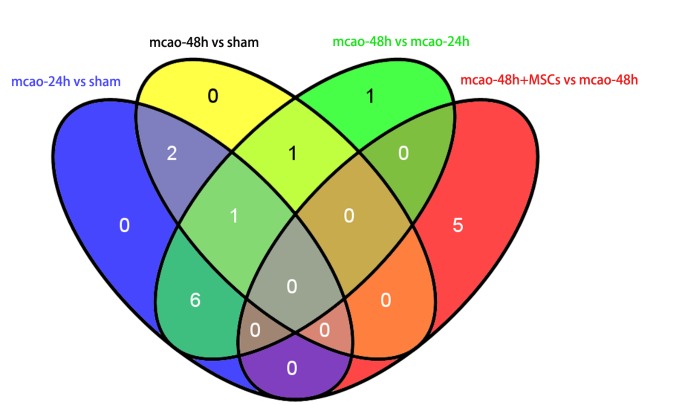
The number of overlapping proteins in the four groups. The blue circle represent the up-regulated and down-regulated proteins at 24 hours after cerebral ischemia compared to sham group, the yellow circle represent the up-regulated and down-regulated proteins at 48 hours after cerebral ischemia compared to sham group, the green circle represent up-regulated and down-regulated proteins at 48 hours after cerebral ischemia compared with the 24 hours after cerebral ischemia group, the red circle represent up-regulated and down-regulated proteins after hUC-MSCs treatment compared with the 48 hours after cerebral ischemia group.

We found that Acat2, Grb2, and Scp2 were down-regulated in the mcao-24h group compared with the sham group and up-regulated in the mcao-48h group compared with the mcao-24h group. On the other hand, Ptgds and Hbb-b1 were up-regulated in the mcao-24h group compared with sham group and down-regulated in the mcao-48h group compared with the mcao-24h group. We also found that Rrbp1, Anxa6, Slk, Nup205 and Psmd6 changed in the mcao-48h + hUC-MSC group only compared with the mcao-48h group. Detailed protein expressions in each group are listed in Table 2. A Venn diagram is used to show the relationship of differentially expressed proteins in these groups (Fig. 1).

**Figure 2.**
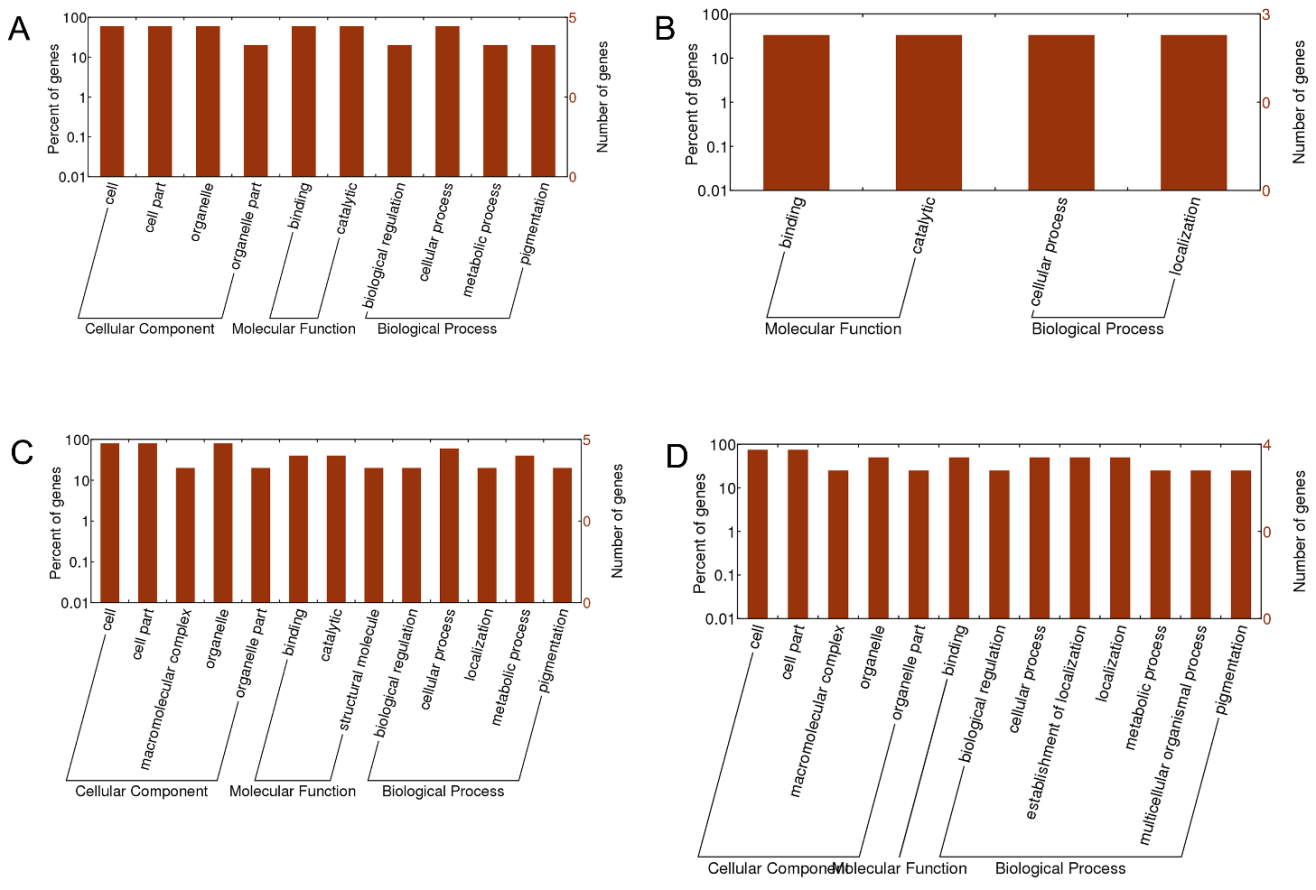
The GO term analysis of differentially expressed proteins in the four experimental groups. The GO term analysis of differentially expressed protein in the mcao-24h/sham group (A), the mcao-48h/sham group (B), the mcao-48h/mcao-24h group (C), and the mcao-48h + MSCs/mcao-48h group (D). Every GO term analysis shows genes involved in cellular component, molecular function and biological process.

### Gene Ontology analysis

In the Gene Ontology (GO) analysis, a gene or protein could be annotated in 3 ways: cellular component, molecular function, and biological process. An analysis of GO was performed to determine the differentially expressed proteins in the four experimental groups. In the mcao-24h/sham group, the differentially expressed proteins were involved in cellular component: cell membrane, cytoplasm, nucleus and organelle; in molecular function: binding and catalytic; and in biological process: biological regulation, cellular process, metabolic process and pigmentation (Fig. 2A). In the mcao-48h/sham group, the differentially expressed proteins were involved in molecular function: binding and catalytic; and in biological process: cellular process and localization (Fig. 2B). In the mcao-48h/mcao-24h group, the differentially expressed proteins were involved in cellular component: cell membrane, cytoplasm, nucleus and organelle; in molecular function: binding, catalytic and structural molecule; and in biological process: biological regulation, cellular process, localization, metabolic process and pigmentation (Fig. 2C). In the mcao-48h + MSCs/mcao-48h group, the differentially expressed proteins were involved in cellular component: cell membrane, cytoplasm, nucleus and organelle; in molecular function: binding; and in biological process: biological regulation, cellular process, establishment of localization, localization, metabolic process, multicellular organismal process and pigmentation (Fig. 2D).

### Network with CD200-CD200R1, CCL21-CXCR3

**Figure 3.**
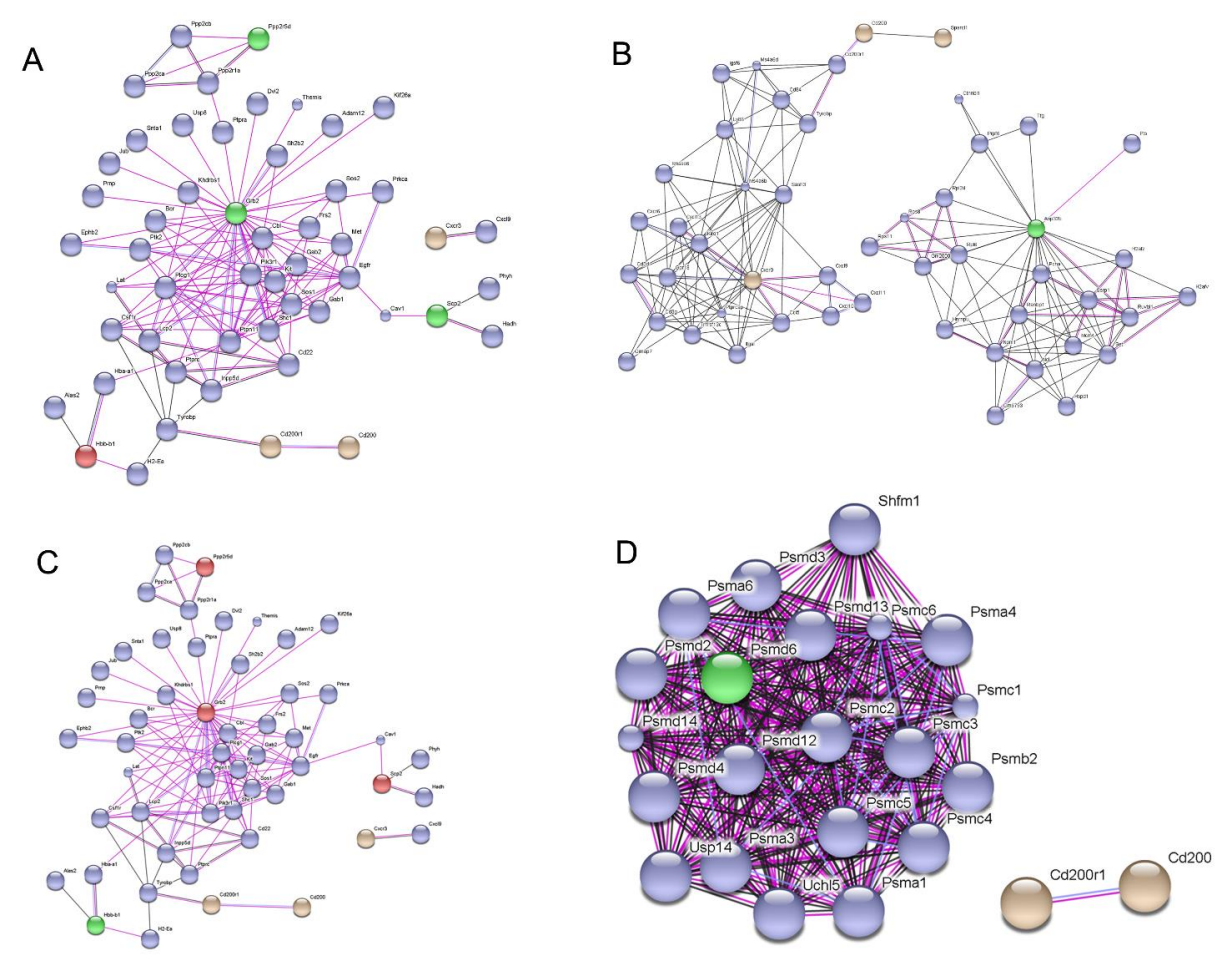
The network associated with CD200-CD200R1, CCL21-CXCR3 of differentially expressed proteins in the four experimental groups. The network with CD200-CD200R1 and CCL21-CXCR3 of differentially expressed proteins in the mcao-24h/sham group (A), the mcao-48h/sham group (B), the mcao-48h/mcao-24h group (C), and the mcao-48h + MSCs/mcao-48h group (D) are shown. Note that the trend in the mcao-24h/sham group was almost opposite to that of the mcao-48h/mcao-24h group.

The analysis of relationship between key molecules associated with neuron-glial crosstalk (CD200-CD200R1, CCL21-CXCR3) and differentially expressed proteins of these four groups are shown in Fig. 3A - 3D.

### Network with Transcription factors

**Figure 4.**
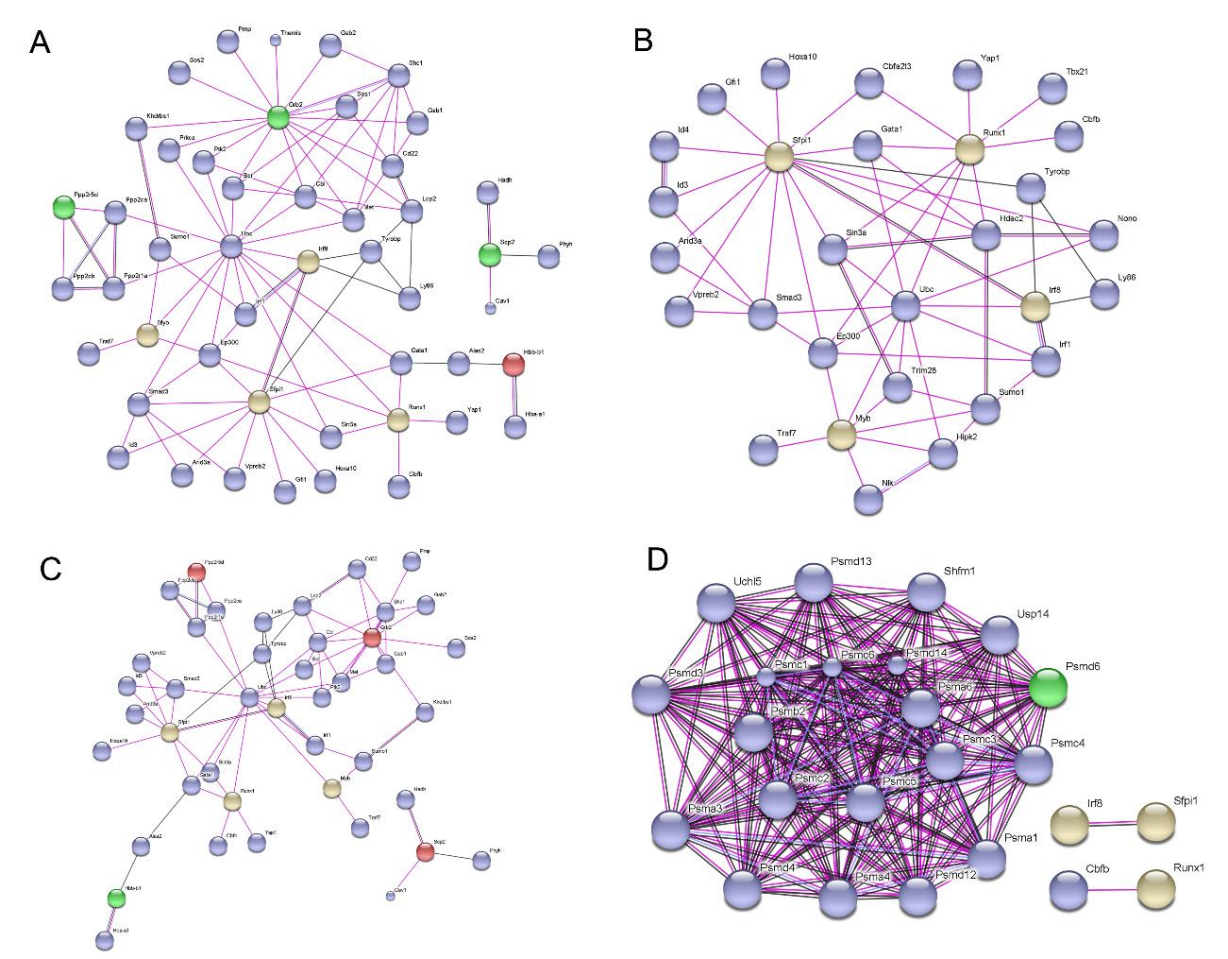
The network with transcription factors (c-myb, Runx-1, Pu.1, Irf8, Hoxb8) of differentially expressed proteins in the four experimental groups. The network with transcription factors (c-myb, Runx-1, Pu.1, Irf8, Hoxb8) of differential expression in the mcao-24h/sham group (A), the mcao-48h/sham group (B), the mcao-48h/mcao-24h group (C) and the mcao-48h + MSCs/mcao-48h group (D) are shown. The transcription factors of c-myb, Runx-1, Pu.1, Irf8, and Hoxb8 are represented by myb, runx1, Spi1, Irf8 and Hoxb8, respectively.

We then analyzed the relationship between transcription factors (c-myb, Runx-1, Pu.1, Irf8, Hoxb8) and differentially expressed proteins in the four groups (Fig. 4A-4D) The transcription factors of c-myb, Runx-1, Pu.1, Irf8, and Hoxb8 are represented by myb, runx1, Spi1, Irf8, and Hoxb8, respectively. We found that Grb2 is located at the center of the network.

### Further verification of the proteomic results

We further confirmed the expressions of Abca13, Grb2, and Ptgds at the mRNA level using qPCR and at the protein level using ELISA.

**Figure 5.**
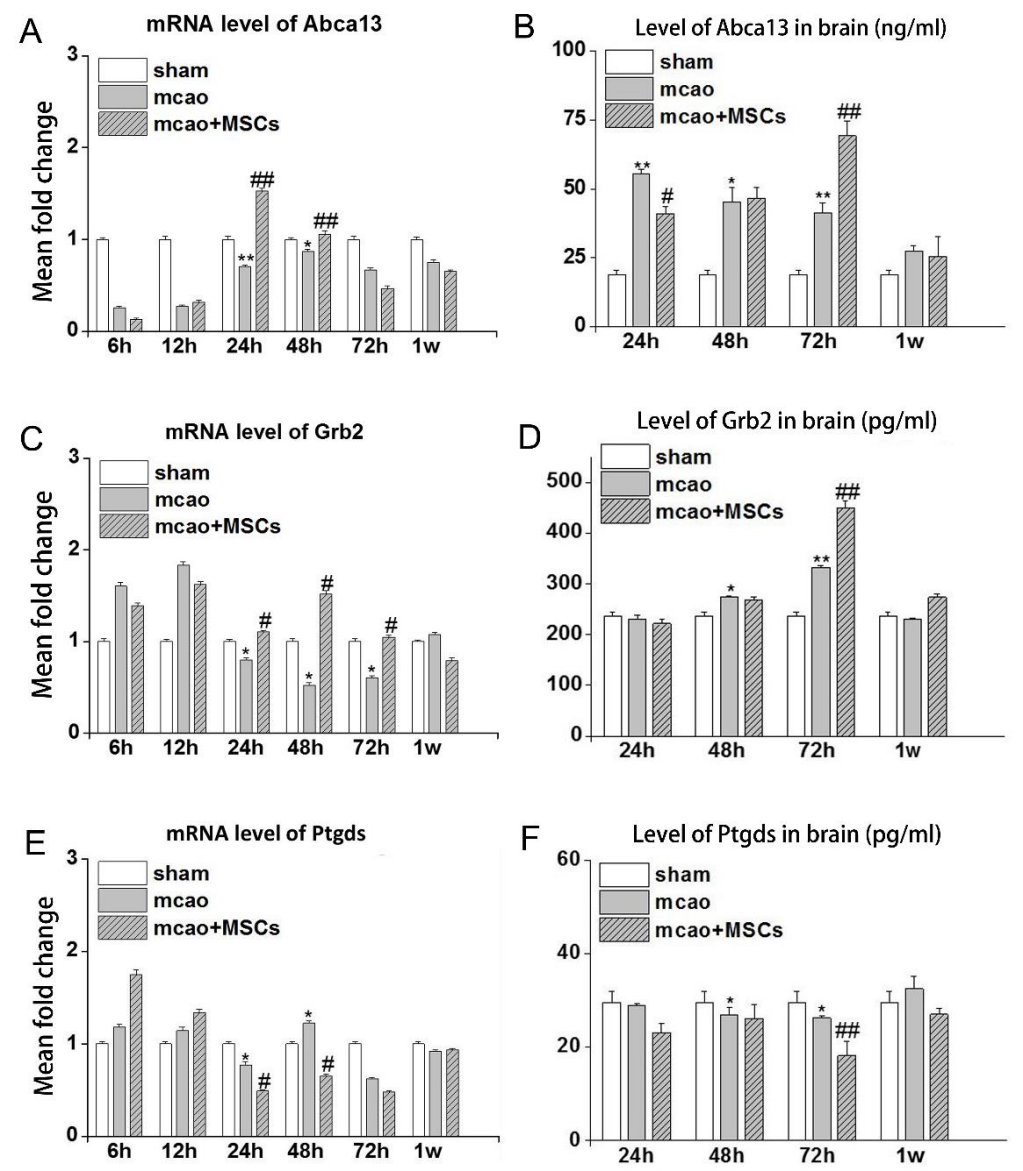
The mRNA and protein levels of Abca13, Grb2, Ptgds in MCAO group and MCAO + MSCs group at indicated time points. The mRNA and protein levels of Abca13 are shown in (A) and (B), respectively. The mRNA and protein levels of Grb2 are shown in (C) and (D), respectively. The mRNA and protein levels of Ptgds are shown in (E) and (F), respectively. *p<0.05; **p<0.01 compared with sham group; #p<0.05; ##p<0.01 compared with MCAO group.

Abca13: At the mRNA level, the Abca13 in the MCAO group was decreased at all time points compared with control group. Abca13 was at its lowest level at 6 h after I/R, and then reached its peak at 48 h after I/R and decreased after 1 week. However, after treatment with hUC-MSCs, Abca13 was increased at 24 h and 48 h after I/R compared with the MCAO group (Fig. 5A). At the protein level, Abca13 was up-regulated in the MCAO group at all the time points compared with the control group. When treated with hUC-MSCs, Abca13 was down-regulated at 24 h and up-regulated at 72 h after I/R compared with the MCAO group (Fig. 5B).

Grb2: At the mRNA level, Grb2 was up-regulated at 6 h and 12 h, but down-regulated at 24 h, 48 h and 72 h after I/R compared with the control group. However, the hUC-MSC treatment could attenuate the changes at all these time points (Fig. 5C). At the protein level, Grb2 was significantly changed only at 72 h after I/R. The protein level of Grb2 in MCAO group was increased compared with the control group, and continually increased after hUC-MSC treatment compared with the MCAO group (Fig. 5D).

Ptgds: At the mRNA level, Ptgds was up-regulated at 6 h, 12 h and 48 h, but down-regulated at 24 h and 72 h after I/R compared with control group. However, the treatment with hUC-MSCs, Ptgds was increased at 6 h, 12 h, 24 h, 72 h, but decreased at 48h after I/R (Fig. 5E). At the protein level, at 72 h after I/R, Ptgds expression in the MCAO group was decreased compared with the control group, which was further decreased after hUC-MSC treatment, compared with MCAO group (Fig. 5F).

## DISCUSSION

In the present study, we analyzed the differential protein expression in the ischemic brain after treatment with hUC-MSCs. A total of 16 proteins were found to be differentially expressed. The function of these proteins were further classified into two categories: 1) differentially expressed proteins during ischemic stroke and 2) differentially expressed proteins after treatment with hUC-MSCs.

### Differentially expressed protein after ischemic stroke

#### Up-regulated proteins

Abca13 and Ptgds were up-regulated after ischemic stroke.

Abca13 is a member of the ABC gene subfamily A (ABCA). Our results showed that Abca13 was up-regulated for up to 72 h after I/R. The expression of Abca13 is elevated in leukemia, prostate tumor, colorectal cancer, and tumor cell lines in central nervous system [16, 17]. Abca13 is thought to be a marker of poor outcome in carcinoma patients [18] as it could play a role in transport of xenobiotics and cause drug resistance. Abca13 was also found to be associated with both schizophrenia and bipolar disorder [19]. There has been no previous report of Abca13’s involvement in stroke.

Ptgds, prostaglandin D synthase, was up-regulated at 24 h, but returned back to its normal level at 48 h after I/R. Ptgds could catalyze the conversion of prostaglandin H2 (PGH2) to prostaglandin D2 (PGD2). PGD2 is known to be involved in smooth muscle contraction/relaxation and modulate the central nervous system, inhibit platelet aggregation [20]. Ptgds is preferentially expressed in the brain, and also regulates non-rapid eye movement sleep [21, 22]. Overexpression of Ptgds is found in patients with attention deficit hyperactivity disorder (ADHD) [23] and multiple sclerosis. One study showed Ptgds might have neuroprotective functions against neuronal death and act as an early stress protein in hypoxic-ischemic encephalopathy [24].

#### Down-regulated proteins

Grb2, Acot11, Acat2, Scp2, Anp32b and Ppp2r5d were down-regulated after stroke.

Grb2 mRNA was downregulated at 24 h, and returned to the normal level at 48 h after I/R. However, ELISA shows that Grb2 protein expression was upregulated at 48 h and 72 h after I/R. Grb2 is a pivotal molecule in signal transduction. In network analysis, there were multiple connections between Grb2 and other proteins. Grb2 links the epidermal growth factor receptor tyrosine kinase and activate the Ras/mitogen-activated protein (MAP) kinase pathway, which subsequently activates ERK1/2 [25-27]. Grb2 also plays a role in lymphocyte development, function, and signaling [28]. Studies have shown that Grb2 overexpressed in breast cancer tissue [29, 30].

Acot11’s down-regulation continued up to 48 h after I/R. Acat2 and Scp2 were down-regulated at 24 h and returned to normal levels at 48 h after I/R. Acat2 and Scp2 are involved in lipid metabolism while Acot11 is involved in obesity [31-34]. Anp32b was down-regulated 48 h after I/R. Anp32b is a substrate for caspase-3 and plays a role in cell apoptosis [35]. There was also a report that Anp32b expression was related to worse outcome in patients with breast cancer [36]. Ppp2r5d was down-regulated 24 h after I/R, and returned to the normal level 48 h after I/R. Ppp2r5d belongs to the phosphatase 2A (PP2A) regulatory subunit B family, and activates PKA-mediated PP2A [37]. One study showed that its expression paralleled protein tau phosphorylation, [38], which is a major component of neurofibrillary tangles in Alzheimer’s disease. Whether these proteins play a role in stroke remain uclear.

### Differentially expressed protein after treatment with hUC-MSCs

#### Up-regulated proteins

Anxa6, Slk, and Rrbp1 were up-regulated after treatment with hUC-MSCs.

Anxa6 was up-regulated significantly after treatment with hUC-MSCs. Anxa6 belongs to a family of calcium dependent protein and has a huge role in physiology and pathology. Anxa6 mediates Ca^2+^ flux across membranes and activate protein kinase C α (PKCα) [39]. In Chlystun’s study, they found that when Anxa6 expression is abolished in the cell, the mitochondria was be fragmented and respiration would be impaired [40]. hUC-MSCs also inhibited apoptosis in stroke. If treatment with hUC-MSCs exerts that function through upregulating Anxa6, then the Anxa6-mediated mitochondria protection may be further explored. Depletion of Anxa6 was also found to be linked to the degradation of activated epidermal growth factor receptor (EGFR) that influences downstream signaling [41]. Anxa6 expression was also inhibited in gastric cancer cells [42, 43]. While elevated Anxa6 expression was found to increase late endosomal cholesterol levels and induce plasma membrane remodelling [44, 45]. There are reports that Anxa6 may play some role in stimulating NF-κB, orchestrating a repair zone when the membrane is injured, and regulate terminal differentiation of chondrocytes [39, 46, 47].

Slk expression was increased significantly after treatment with hUC-MSCs. Slk plays important roles in many physiological and pathological processes. In the brain, Slk is preferentially expressed in adult neuronal cells, but not in resting astrocytes or microglia. In Zhang’s study, Slk-expressing neuronal cells were decreased, while Slk was up-regulated in other cell types after MCAO [48]. These Slk expressing cells may be infiltrated leukocytes or activated native microglia. Overexpression of Slk could induce apoptosis either through a p38 or c-Jun N-terminal kinase-1 (JNK1)-dependent pathway [49-51]. In breast cancer cells, Slk could activate HER2/Neu/ErbB2 for driving chemotaxis. Slk is also required in cell migration and cell cycle progression through G2 [52, 53]. In addition, Slk was found to be critical in embryonic development, hypertension, and tissue injury [49].

After treatment with hUC-MSCs, Rrbp1 was significantly increased. Rrbp1 is a protein in the endoplasmic reticulum (ER) membrane, which binds with ribosomes. Overexpression of Rrbp1 was found in patients with lung, colorectal, and breast cancers, of which predict an undesirable outcome [54-56]. It might inhibit apoptosis by alleviating ER stress in cancer cells. However, the role of Rrbp1in stroke is unclear.

#### Down-regulated proteins

Psmd6 and Nup205 were down-regulated after treatment with hUC-MSCs.

Psmd6 is a member of the protease subunit S10 family involved in the degradation of ubiquitinated proteins and DNA repair [57]. Studies on Nup205 are limited [58, 59]. None of them have been reported to be involved in stroke.

In this study, the mass spectrometer was used to determine the protein expression level. The information gathered from that was analyzed by software to determine differentiatal expression of proteins. We performed qPCR and ELISA to confirm proteomic results.

Abca13 is a member of ABC gene subfamily A (ABCA), which is an important member of the family in pathologic and physiologic processes in the human body. Grb2 plays an important role in signal transduction which involves in multiple pathways. Ptgds is able to catalyze the production of prostaglandin D2, which regulates a number of aspects in central nervous system. Because of their critical roles in pathology and physiology, we further studied these 3 proteins at the mRNA and protein levels at several time points. Our results showed that the Abca13 protein expression was consistent with the proteomic data, but not at the mRNA level. While the Grb2 expression at the protein level was inconsistent with the proteomic results, the trend of the mRNA expression pattern was similar. However, Ptgds expression at both the protein and mRNA levels were inconsistent with the proteomic data. Taken together, the data suggest that Abca13 and Grb2 may be more important in ischemic stroke after hUC-MSC transplantation.

The results from the proteomic study showed 16 differentially expressed proteins. We chosed three of them to validate their expressions, and found that Abca13 and Grb2 could be of further interest.

There are some limitations regarding the present research. Protein validation was not conducted for each of the differentially expressed proteins. Hence, proteins that may be of interest for the treatment of stroke were not identified yet. Proteomic results for Abca13, Grb2 and Ptgds showed that Abca13 at the protein level and Grb2 at the mRNA level were consistent with the proteomic study; their functions will be determined in the future.

## Conclusion

In the present study, we analyzed the expression profiles of proteins after stroke in the presence or absence of hUC-MSCs treatment using iTRAQ. We identified 16 differentially expressed proteins. There were complicated connections between them and transcription factors or other proteins. Proteomic validation results showed that Abca13 and Grb2 are of interest and could be further pursued. Many of these proteins that are highlighted in this study have not been reported in the field of stroke before. We believe the proteomic method used in the present study could provide clues for the involvement of these proteins in stroke, which could help us further understand stroke and facilitate treatment with hUC-MSCs for ischemic stroke patients.
